# Degenerate intervertebral disc‐like pH induces a catabolic mechanoresponse in human nucleus pulposus cells

**DOI:** 10.1002/jsp2.1004

**Published:** 2018-03-11

**Authors:** Nathan W. Hodson, Sonal Patel, Stephen M. Richardson, Judith A. Hoyland, Hamish T. J. Gilbert

**Affiliations:** ^1^ Division of Cell Matrix Biology and Regenerative Medicine, School of Biological Sciences, Faculty of Biology, Medicine and Health University of Manchester Manchester UK; ^2^ NIHR Manchester Musculoskeletal Biomedical Research Unit, Central Manchester NHS Foundation Trust Manchester Academic Health Science Centre Manchester UK; ^3^ Wellcome Trust Centre for Cell‐Matrix Research, Division of Cell Matrix Biology and Regenerative Medicine, School of Biological Sciences, Faculty of Biology, Medicine and Health University of Manchester Manchester UK

**Keywords:** acidic pH, intervertebral disc degeneration, mechanical load, mechanotransduction, nucleus pulposus

## Abstract

Mechanical stimulation is known to influence intervertebral disc (IVD) cell behavior and function, but the effect on disc cells is routinely considered in isolation from other microenvironmental factors. Acidic pH has been shown to be a prominent and detrimental microenvironmental factor present in degenerate IVDs, but its influence on the human disc cell mechanoresponse has never been studied. We investigated the response of agarose‐encapsulated human nucleus pulposus (NP) cells to 0.004 MPa, 1.0 Hz and 1 hour of compression (Flexcell FX4000 Compression System) under pH conditions representative of nondegenerate (pH 7.1) and degenerate (pH 6.5) IVDs. Cell viability, extracellular matrix production, and expression of anabolic/anti‐catabolic and catabolic genes were assessed. We report that preculture of NP cells in agarose gels was required in order for cells to be mechanoresponsive, and this correlated with increased type VI collagen, α5β1 integrin, and fibronectin expression. Furthermore, the matrix homeostatic response observed at pH 7.1 (representative of nondegenerate IVDs; increased aggrecan [AGC], tissue inhibitor of metalloproteinases‐1 [TIMP1], matrix metalloproteinase‐3 [MMP3], a disintegrin and metalloproteinase with thrombospondin motif‐5 [ADAMTS5] gene expression) was RGD‐integrin dependent, whereas only MMP3 remained mechanoresponsive at pH 6.5, and this was independent of RGD‐integrins. Our findings suggest differential mechanotransduction pathways operating for specific genes, with RGD‐integrin dependent AGC expression, but not RGD‐independent MMP3 expression, inhibited at pH representative of degenerate IVDs (pH 6.5), which could contribute to the catabolic phenotype observed during IVD degeneration.

**Clinical significance:**

Characterizing the influence of the mechanical and chemical intervertebral disc microenvironment on disc cells, particularly in disc degeneration, could help develop future therapeutic strategies for the treatment of discogenic back pain.

## 1 INTRODUCTION

The intervertebral disc (IVD) is a fibrocartilage pad located between motion segments in the spine. The healthy IVD facilitates flexion and rotation of the spine through all planes while maintaining stability^1^ and is predicted to experience mechanical compressive loads in excess of 2.3 MPa (depending on the activity).^2^, ^3^ The mechanical compressive forces are transduced to the resident cells, both directly through the coupling of cell surface receptors and the extracellular matrix (ECM) and indirectly through compression‐induced changes to the ECM, including changes in osmotic pressure, fixed charge density, and extracellular pH.^4^ The pericellular matrix, characterized as a type VI collagen and perlecan‐rich matrix immediately proximal to the cell,^5^, ^6^ has been shown to play an important role in transducing mechanical force from the surrounding tissue to the cell surface.^7^

Previous studies have shown that the type, magnitude, frequency, and duration of mechanical stimuli are all important parameters in influencing the mechanoresponse of disc cells.^8^, ^9^, ^10^, ^11^, ^12^, ^13^, ^14^, ^15^, ^16^, ^17^ Interestingly, there appears to be a mechanical force threshold whereby disc cells will respond to the stimulus by increasing the expression of anabolic matrix factors, whereas stimuli above or below this range results in matrix catabolism.^8^, ^12^, ^14^, ^15^ Furthermore, the response of IVD cells to mechanical stimuli has been reported to differ depending on whether cells are derived from healthy or diseased tissue.^8^, ^18^, ^19^ Understanding how disc cells respond to mechanical stimuli during pathological conditions may increase our understanding of degenerative disc disease and lead to novel therapeutic strategies for the treatment of the associated back pain.

When considering the effect of mechanical stimuli on disc cells, it is important to consider other microenvironmental factors that influence disc cells in vivo. The IVD microenvironment is a challenging and complex niche, with low levels of oxygen and glucose and high levels of CO_2_, lactate, osmolarity, and mechanical loads.^20^, ^21^ Furthermore, this niche becomes more hostile with the progression of IVD degeneration, driven by a reduction in diffusion coefficients, which lead to less nutrients and more lactate within the center of the disc.^22^, ^23^ The pH of the IVD has been shown to range from pH 7.1 in healthy tissues to pH 6.8 in degenerate tissue, with values as low as pH 5.5 recorded in severely degenerated discs.^24^, ^25^ We have recently demonstrated that an acidic pH promotes induction of a degenerate phenotype in nondegenerate human nucleus pulposus (NP) cells, including increased cell death, increased expression of inflammatory cytokines, neurotrophins, and matrix‐degrading enzymes and decreased matrix synthesis.^26^

To date, the majority of IVD mechanical loading studies have been conducted under standard culture conditions at pH 7.4, which fail to mimic the IVD microenvironment. A study by Neidlinger‐Wilke et al, however, reported the effect of changes in osmolarity, glucose, and oxygen concentrations, as well as changes in pH on the mechanoresponse of bovine NP cells to hydrostatic pressure.^27^ Under varying pH (pH 7.2, 6.8, and 6.5), the authors observed differential gene expression responses to mechanical loading, most notably a decrease in aggrecan (AGC) following mechanical stimulation at pH 7.2, but an increase in AGC expression with mechanical load at pH 6.8 and 6.5.^27^

While this raises interesting questions regarding the response of bovine NP cells,^27^ there have been no studies assessing the response of human IVD cells to both mechanical stimulus and changes in pH. We aimed, therefore, to assess the response of human NP cells to physiological compression under pH conditions representative of healthy, nondegenerate IVDs (pH 7.1) and degenerate IVDs (pH 6.5) in the context of matrix homeostasis (ie, assessing 2 important genes involved in matrix anabolism (AGC and tissue inhibitor of metalloproteinases‐1 [TIMP1]) and matrix catabolism (matrix metalloproteinase‐3 [MMP3] and a disintegrin and metalloproteinase with thrombospondin motifs‐5 [ADAMTS5]). In addition to assessing the mechanoresponse of NP cells to compression at pH representative of IVDs in health and disease, we investigated the mechanotransduction pathways operating in NP cells under these conditions. Increasing our understanding of how disc cells respond to mechanical stimuli both in health and disease will help further our knowledge of the pathophysiology of IVD degeneration and potentially lead to the identification of novel therapeutic targets for its treatment.

## 2 METHODS

### 2.1 IVD tissue

Human IVD tissue was collected from cadavers (within 24 hours of death) (2 males aged 36 and 63) and from a patient undergoing lumbar spinal surgery following trauma to the spine (female aged 47), with patients’ or relatives’ written informed consent and Research Ethics Committees approval (National Research Ethics Service Committee North West), with all methodology performed in accordance with the Committee's guidelines. Tissue from 1 disc per donor was processed for cell extraction, and representative samples of all tissues containing intact AF and NP regions were formalin‐fixed, paraffin‐embedded, and sections histologically graded as previously reported.^28^ Cadaveric and surgical tissue was histologically graded as 0‐3 (nondegenerate) and were obtained from individuals with no documented history of back pain.

### 2.2 Isolation and culture of NP cells

NP tissue (excluding outer and inner AF) was separated from the IVD within 24 hours of death or surgical removal and finely minced prior to enzymatic digestion as previously reported.^28^ NP cells were cultured in standard medium (Dulbecco's modified Eagle's medium [DMEM] with glucose 4.5 g/L, l‐alanyl‐glutamine [Sigma‐Aldrich, Poole, UK] containing 100 mM sodium pyruvate, 10 μM ascorbate‐2‐phosphate, 250 ng/mL amphotericin, 100 U/mL penicillin, 100 μg/mL streptomycin [Invitrogen, Paisley, UK], and 10% fetal calf serum [Life Technologies, Paisley, UK]) and expanded in monolayer, with medium changed every 2‐3 days. Subconfluent NP cells with passage numbers of ≤4 were trypsinized (Life Technologies) and encapsulated into 2% low gelling temperature agarose gel (Sigma) as detailed previously.^29^ Briefly, 5× DMEM (prepared from powder (DMEM:F‐12 (1:1) [Fisher Scientific]) was mixed with molten agarose gel (2.5%) and dissolved in dH_2_O to give a final agarose gel solution of 2% in 1× DMEM. Molten agarose gel was maintained at 38°C to prevent polymerization, and NP cells were resuspended at a final concentration of 2 × 10^6^/mL. The cell agarose solution was pipetted into 24‐well plates (350 μL/well) and allowed to solidify at room temp for 15 minutes, and cylinders of 15 mm diameter were cored and placed into BioPress (Flexcell International, Burlington, North Carolina) culture plates. Standard medium was added to the BioPress plate, ensuring the agarose construct was fully submerged, and the constructs were cultured under standard conditions for either 1 or 7 days (medium changed every 2‐3 days).

### 2.3 Preparation of pH‐modified medium

pH‐adjusted medium was prepared as previously described.^26^ Briefly, a basal medium was prepared from DMEM/F‐12‐powdered medium without sodium bicarbonate (Fisher Scientific), and the following supplements were added: 50 μg/mL ascorbic acid, 250 ng/mL amphotericin, 100 U/mL penicillin, 100 μg/mL streptomycin (Invitrogen), and 10% fetal calf serum (Life Technologies). The correct amount of sodium bicarbonate was added using the Henderson‐Hasselbalch equation, which—for the purpose of sodium bicarbonate buffered culture medium—can be expressed as pH = 6.1 + log (52 [mg/mL NaHCO_3_/% CO_2_] − 1).^30^ HCl and NaOH were used to pH the medium to the desired values (pH 7.1 and 6.5), and the medium was allowed to equilibrate overnight in a 5% CO_2_ incubator at 37°C. The pH of the medium was confirmed a second time, and the medium was sterilized using a 0.04‐μm filter and stored at 4°C until needed (medium not stored for longer than 1 week).

### 2.4 Viability assay

NP cells in 2% agarose gels were stained with LIVE/DEAD viability stain (Life Technologies L3224) as per the manufacturer's instructions, and multiple fields of view were imaged using a Leica SP5 upright confocal microscope with dipping lens. Green staining identified viable cells, while nonviable cells were red or had red nuclei with green cytoplasm. Encapsulated chondrocytes have been reported to remain viable in a 3% agarose gel with a thickness of 3 mm cultured for 9 days, with viability unaffected by spatial distribution.^31^ The agarose constructs in this study had a thickness of 1.5 mm in order to minimize any diffusion‐related effects on viability.

### 2.5 Immunohistochemistry (IHC)

Encapsulated NP cells in agarose gels were cultured in standard medium for 1 or 7 days, immediately fixed in 4% paraformaldehyde (PFA) for 20 minutes at room temp, or cultured for a further 24 hours in pH‐adjusted medium (pH 7.1 or 6.5) and then fixed. Fixed samples were processed into paraffin wax, sectioned (5 μm sections), and then mounted onto glass slides for antibody staining/hematoxylin and eosin staining, as previously reported.^28^ Briefly, mounted sections were dewaxed with xylene, rehydrated, and treated with 0.01% α‐chymotrypsin from bovine pancreas (Sigma) for antigen retrieval. Endogenous peroxidase activity was blocked using hydrogen peroxide 30% w/v (Fisher Scientific, H/1750/117) and 25% normal goat serum (Sigma), and 1% BSA (Sigma) was used to prevent nonspecific binding of antibodies. Sections were incubated with antibodies against type VI collagen (Developmental Studies Hybridoma Bank, Lowa City, Iowa, 5C6), α5 integrin subunit Millipore (Merck, Nottingham, UK, MAB1956) (1:250), β1 integrin subunit (Merck, MAB1965) (1:250), α5β1 integrin heterodimer (Merck, MAB1969) (1:250), or fibronectin (Pierce Antibodies, MA5‐11981) (1:250) for an hour at room temperature. Following 3× washes in Tris‐buffered saline (TBS), samples were incubated with biotinylated secondary antibodies (goat anti‐mouse, Santa Cruz Biotechnology, SC3795; or goat anti‐rabbit, Santa Cruz Biotechnology, SC3840) at room temp for 1 hour. Following a further 3× washes in TBS, antibody binding was visualized using the streptavidin‐biotin complex (Dako) technique with 3,3′‐diaminobenzidine tetrahydrochloride solution (Sigma), and sections were counterstained using hematoxylin (Surgipath, Peterborough, UK). Multiple fields of view were imaged, with representative images presented. Staining appeared similar across the constructs (ie, unaffected by spatial distribution of cells).

### 2.6 Compression of agarose‐encapsulated NP cells at pH representative of nondegenerate and degenerate IVDs

Compression of precultured (1 or 7 days preculture in standard medium followed by a subsequent 24 hours preculture at a pH representative of either nondegenerate [pH 7.1] or degenerate [pH 6.5] IVDs) agarose‐encapsulated NP cells was administered using a Flexcell FX4000 Compression System (Flexcell International). A physiologically relevant mechanical compression regime of 0.004 MPa compression (~14% compressive strain) at a frequency of 1.0 Hz for 1 hour was used. Physiological compression of intact human IVD motion segments (bone‐disc‐bone) has been previously shown to result in axial compressive strains of up to 9.9%,^32^ with high shear peak strains of up to 26%.^33^ The proportion of compressive strains likely transduced from tissue to resident cells remains unknown but is predicted to be somewhat less than the strain experienced by the tissue. Similarly, the amount of strain experienced by the cells in our study remains unknown but is likely to be less than the strain experienced by the agarose gel, suggesting its physiological relevance.

The involvement of RGD‐recognizing integrins was assessed using RGD function‐blocking peptides as described previously.^19^ Briefly, encapsulated cells (1 or 7 days preculture in standard medium followed by 24 hours culture in medium at pH 7.1 or 6.5) were cultured with and without control RAD peptides (100 μg/mL) (Calbiochem, Cat no. 03‐34‐0052) or function‐blocking RGD peptides (100 μg/mL) (Calbiochem, Cat no. 03‐34‐0035) for 1 hour prior to mechanical compression.

### 2.7 Quantitative real‐time polymerase chain reaction (QRT‐PCR)

Total RNA was extracted from cells immediately following 1 hour of compression, with relevant time‐matched uncompressed control cells extracted in parallel, using a combination of a gel extraction kit (QIAquick Gel Extraction Kit, Qiagen, Manchester, UK) and RNA extraction kit (RNeasy Mini Kit, Qiagen) as previously described.^29^

Choosing an appropriate time point for monitoring of the mechanoresponse is important in order to ensure that a cellular response can be measured. Different aspects of cellular metabolism will be effected at different times following a dose of mechanical input. For example, changes in gene expression will occur much faster than changes in protein expression. Extraction of RNA from chondrocytes immediately following 30 minutes of compression demonstrated changes in early response genes.^29^ We have previously reported changes in matrix‐associated genes following an hour of rest after the administration of 20 minutes of cyclic tensile strain to human AF cells (80 minutes in total from the beginning of strain cycle),^8^ and as such, in this study, we chose to lyse cells for mRNA analysis immediately following the 1‐hour compression regime to observe immediate changes in mRNA in response to load.

Briefly, for extraction of RNA from 1 agarose/cell construct, 0.75 mL QG buffer from the gel extraction kit was mixed with 1 mL RNeasy lysis buffer (supplemented with 2‐mercaptoethanol as per manufacturer's instructions [Qiagen]) and an agarose/cell construct submerged into the mixture immediately after mechanical loading. Constructs were dissolved in this buffer at room temperature for 10 minutes (or until no trace of agarose gel remained). A total of 1 mL of 70% ethanol was added to each sample, the sample was loaded into an RNeasy spin column, and RNA was extracted as per manufacturer's protocol (including on‐column DNase treatment).

RNA quality and quantity were determined using the Nanodrop ND‐1000 Spectrophotometer (Nanodrop Technologies, Wilmington, UK), and 300 ng of RNA was reverse transcribed using the High Capacity Reverse Transcription Kit (Applied Biosystems, Warrington, UK). QRT‐PCR was performed in triplicate using LuminoCt qPCR ReadyMix (Sigma) with primers and probes (Sigma) for mitochondrial ribosomal protein L19 (MRPL19) forward primer (F)‐CCACATTCCAGAGTTCTA, reverse primer (R)‐CCGAGGATTATAAAGTTCAAA, probe (P)‐CAAATCTCGACACCTTGTCCTTCG; eukaryotic translation initiation factor 2B subunit alpha (EIF2B1) F‐TCCCAGATAAGTTTAAGTATAAG, R‐AGCAGAGTGATTAAGGAA, P‐CGCAGACTGGACAAGACCTCA; AGC F‐GGCTTCCACCAGTGTGAC, R‐GTGTCTCGGATGCCATACG, P‐TGACCAGACTGTCAGATACCCCATCCA; MMP3 F‐GTGGAGTTCCTGATGTTG, R‐GCATCTTTTGGCAAATCTG, P‐AATTCACAATCCTGTATGTAAGGT; and a disintegrin and metalloproteinase with thrombospondin motifs ADAMTS5 F‐CGCTTAATGTCTTCCATCCTTA, R‐GGATCTGCTTTCGTGGTAG, P‐CAGCAAACAGTTACCATGGCCATCATC; and TIMP1 F‐GACACCAGAGAACCCA, R‐GACGAGGTCGGAATTG, P‐CTGGCTTCTGGCATCCT. Data were analyzed using the 2^−∆Ct^ method^8^, ^34^ and normalized to the endogenous control genes MRPL19 and EIF2B1.

### 2.8 Statistical analysis

All experiments were conducted using 3 agarose gels containing encapsulated cells derived from 3 individual human donors. Each gel was considered separately, with the mean ± SEM calculated for each treatment group. Each effect was considered in isolation using a Mann‐Whitney *U* test (data determined as nonparametric using D'Agostino‐Pearson normality test) with differences between treatments deemed significant if *P* ≤ .05.

## 3 RESULTS

### 3.1 Viability of encapsulated human NP cells remained high

**Figure 1:**
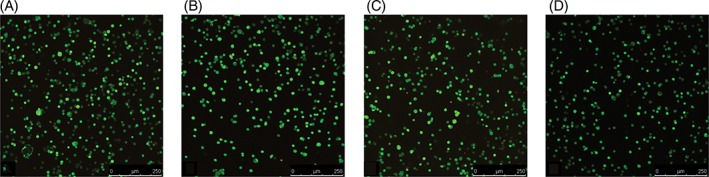
Live/dead staining of human nucleus pulposus (NP) cells encapsulated (2 × 10^6^ cells/mL) in 2% agarose gels and cultured for 7 days in standard Dulbecco's modified Eagle's medium (DMEM) at pH 7.4. Encapsulated cells were then cultured for 24 hours at either pH 7.1 or 6.5 (representative of nondegenerate and degenerate intervertebral discs (IVDs), respectively) and either compressed (0.004 MPa at 1.0 Hz) or not for 1 hour. Cell viability remained high, ≥90% (indicated by green cells), with levels of cell death (indicated by red cells) similar across all treatments. (A) Unloaded gel at pH 7.1. (B) Mechanically stimulated (MS) gel at pH 7.1. (C) Unloaded gel at pH 6.5. (D) MS gel at pH 6.5

Cell viability remained high (>90%) following encapsulation of human NP cells in 2% agarose gel and cultured for 7 days in standard medium at pH 7.4, followed by 24‐hour culture in a medium of either pH 7.1 (Figure 1A) or pH 6.5 (Figure 1C). Compression of agarose/cell constructs with 0.004 MPa compression at 1.0 Hz for 1 hour did not affect viability (compression at pH 7.1) (Figure 1B) and pH 6.5 (Figure 1D), which remained high (>90%).

### 3.2 The mechanoresponse of NP cells in agarose gels is preculture duration dependent and altered by acidic pH, leading toward a more catabolic phenotype

**Figure 2:**
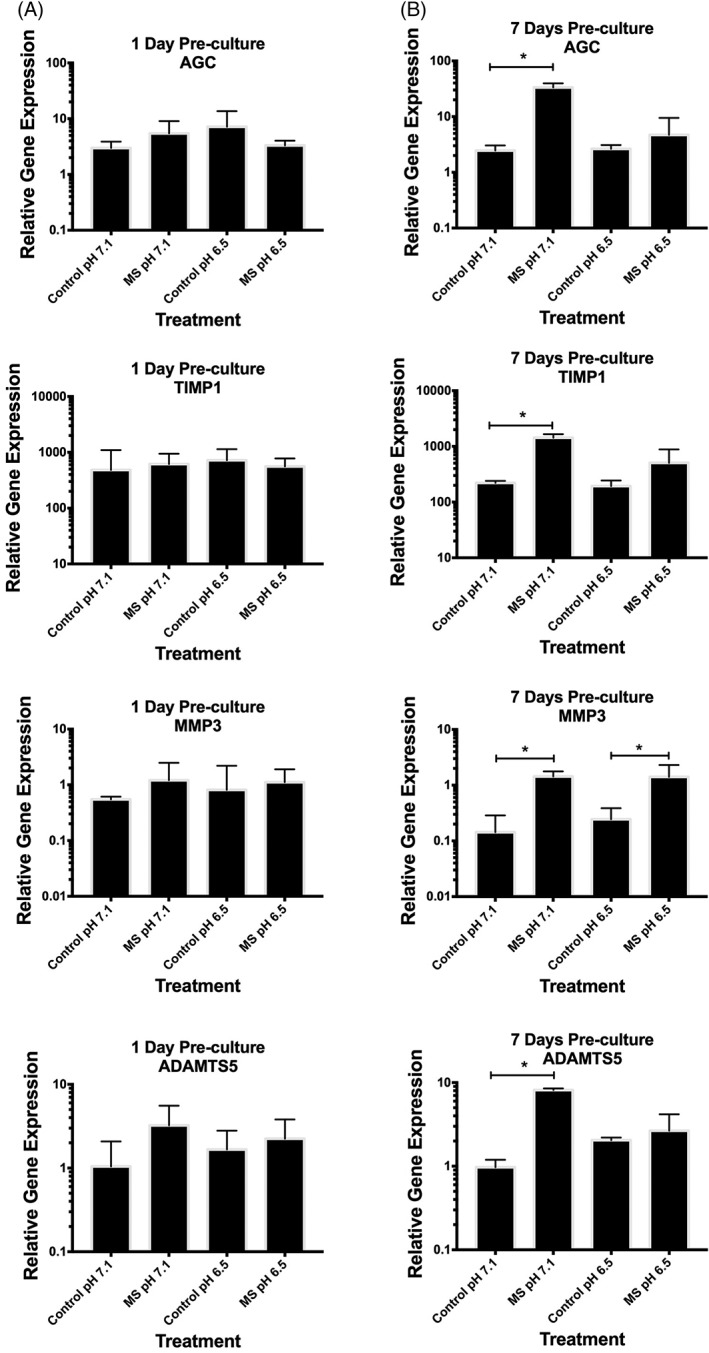
Nucleus pulposus (NP) cells (*n* = 3) were encapsulated (2 × 10^6^ cells/mL) in 2% agarose gel and cultured for (A) 1 day or (B) 7 days in standard Dulbecco's modified Eagle's medium (DMEM) medium. Encapsulated cells were then cultured for a further 24 hours in DMEM at pH 7.1 or 6.5 (to mimic the nondegenerate or degenerate intervertebral disc [IVD] microenvironment, respectively) and then mechanically compressed with 0.004 MPa at 1.0 Hz for 1 hour. Uncompressed encapsulated cells served as the control. Gene expression is presented using the 2‐dCt method^34^ relative to MRPL19 and EIF2B1. (A) Compression of encapsulated cells following 1 day of preculture in standard DMEM resulted in no change to the expression of any genes assessed, at either pH 7.1 or 6.5. (B) However, both anabolic/anti‐catabolic genes (aggrecan [AGC] and tissue inhibitor of metalloproteinases‐1 [TIMP1]) increased in NP cells compressed at pH 7.4, but not at pH 6.5, following 7 days of preculture in standard medium. The catabolic genes (matrix metalloproteinase‐3 [MMP3] and a disintegrin and metalloproteinase with thrombospondin motifs‐5 [ADAMTS5]) both increased in NP cells precultured for 7 days in standard medium and then compressed at pH 7.1. Interestingly, MMP3 gene expression was also increased in NP cells compressed at pH 6.5. The Mann‐Whitney *U* test was used to test for significance between control and compressed treatments, with *P* ≤ .05 considered significant and indicated by “*”

Encapsulated NP cells did not alter their gene expression in response to compression following 1 day of preculture in standard medium (pH 7.4) at either pH tested (pH 7.1 or 6.5) (Figure 2A). However, following 7 days of preculture, mechanically compressed encapsulated NP cells significantly increased their gene expression of all genes assessed (anabolic/anti‐catabolic genes AGC [13‐fold, *P* = .03] and TIMP1 [6.5‐fold, *P* = .02] and catabolic genes MMP3 [10‐fold, *P* = .04] and ADAMTS5 [8‐fold, *P* = .04]) when stimulated at pH 7.1 (representative of the pH of a nondegenerate IVD) suggestive of matrix homeostasis (Figure 2B). Importantly, when encapsulated NP cells were compressed at a pH representative of a degenerate IVD (pH 6.5), cells significantly increased expression of MMP3 (6‐fold, *P* = .04) only, suggestive of a shift toward a more catabolic phenotype (Figure 2B). There was no significant difference in the gene expression of any genes assessed in uncompressed encapsulated NP cells cultured at pH 7.1 and 6.5 (Figure 2).

### 3.3 Mechanically induced increased expression of AGC, but not MMP3, in agarose encapsulated NP cells is dependent on RGD‐recognizing integrins

**Figure 3:**
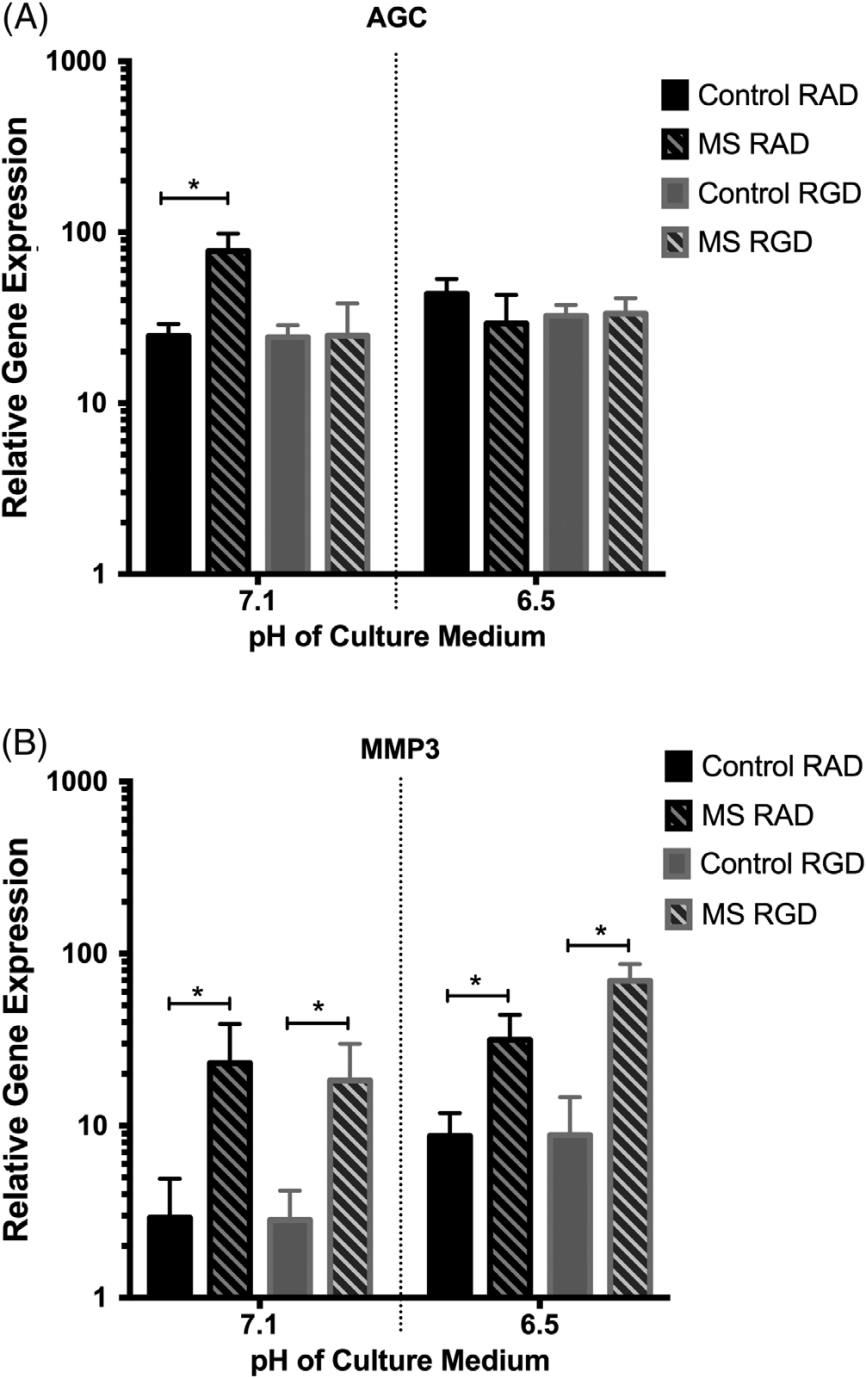
The relative gene expression of (A) aggrecan (AGC) and (B) matrix metalloproteinase‐3 (MMP3) in nucleus pulposus (NP) cells (*n* = 3) following 7 days preculture in standard medium and compressed in agarose gels at pH 7.1 and 6.5, with small peptide molecules (RAD nonblocking control peptides and RGD function‐blocking peptides—which block RGD‐recognizing integrins) was assessed. **(A)** AGC gene expression increased in NP cells in the RAD control peptide group following compression at pH 7.1 but not at pH 6.5. The compression‐induced increase in AGC at pH 7.1 was found to be RGD‐integrin dependent, suggested by the loss of AGC mechanosensitivity when cells/gels were precultured with RGD peptides prior to compression. (B) MMP3 gene expression increased in RAD peptide‐treated NP cells following compression at pH 7.1 and 6.5. RGD peptides had no effect on the mechanosensitivity, suggesting MMP3 mechanoregulation occurs independently from RGD‐recognizing integrin involvement. The Mann‐Whitney *U* test was used to test for significance between control and compressed treatments, with *P* ≤ .05 considered significant and indicated by “*”

One anabolic (AGC) and one catabolic (MMP3) gene were selected to move forward to investigate the mechanotransduction pathways operating during the mechanoexpression of these genes at different pH. When NP cells, following 7 days of preculture in standard medium, were cultured in the presence of RAD peptides (an amino acid chain that is not recognized by integrin receptors) and mechanically compressed at pH 7.1 (pH similar to that recorded in nondegenerate discs), AGC gene expression was increased (3‐fold, *P* = .04) compared to uncompressed controls (Figure 3A). However, when NP cells were compressed in the presence of RGD peptides (known to interact with RGD‐recognizing integrins, including the fibronectin receptor α5β1) at an identical pH (pH 7.1), AGC gene expression was prevented from increasing, with expression levels remaining similar to uncompressed controls (Figure 3A). AGC gene expression was not mechanoresponsive at pH 6.5 (similar to that reported in Figure 2B) (Figure 3A). Compression of NP cells, following 7 days of preculture in standard medium, at pH 7.1 and 6.5, in the presence of control RAD peptides, led to an increase in MMP3 expression (8‐fold increase, *P* = .03, at pH 7.1 and 3.5‐fold increase, *P* = .04, at pH 6.5) compared to uncompressed controls (Figure 3B), similar to that reported in Figure 2B. However, unlike that observed with AGC gene expression at pH 7.1, the increase in MMP3 expression did not appear to be RGD integrin dependent, with RGD function‐blocking peptides unable to prevent the mechanically induced increase in MMP3 (6.5‐fold, *P* = .02, at pH 7.1 and 8‐fold, *P* = .03, at pH 6.5) at either pH tested (Figure 3B).

### 3.4 Agarose‐encapsulated NP cells express type VI collagen and α5β1 integrins following 7 days, but not 1 day, of preculture in standard medium

**Figure 4:**
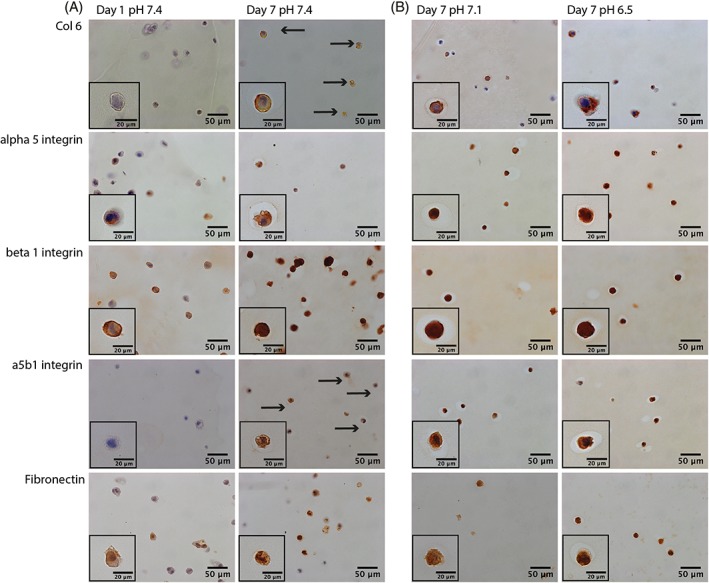
Immunohistochemistry of agarose gels following: (A) 1 and 7 days of culture in standard medium at pH 7.4 and (B) a subsequent 24 hours at pH 7.1 or 6.5. Nucleus pulposus (NP) cells/agarose gels were snap frozen in liquid nitrogen and cryosectioned. Antibodies against the pericellular marker type VI collagen, the integrin receptor α5β1, and separate subunits α5 and β1 and the extracellular matrix protein fibronectin (the predominant ligand of α5β1 integrins) were used to probe sections. (A) NP cells did not appear to express type VI collagen or α5β1 integrin following 1 day of preculture. However, NP cells did express both type VI collagen and α5β1 following 7 days of preculture (black arrows indicate cells expressing proteins which were not expressed at day 0), (B) with 24 hours of culture at pH 7.1 or 6.5 not affecting this expression. (A) NP cells did express the α5 and β1 integrin subunits, as well as fibronectin, following 1 and 7 days of preculture in standard medium, (B) with culture at pH 7.1 or 6.5 having no noticeable affect on the expression

NP cells demonstrated no signs of positive staining for the pericellular marker, type VI collagen, or the fibronectin‐binding receptor, α5β1 integrin, following 1 day of preculture in agarose gels (Figure 4A). However, on 7 days of preculture in standard medium at pH 7.4, NP cells stained positively for both type VI collagen and α5β1 integrin heterodimer (Figure 4A). Positive staining for both the α5 and β1 integrin monomer subunits, and fibronectin, was detected in NP cells following both 1 and 7 days of preculture in agarose gels (Figure 4A).

### 3.5 Culture of agarose‐encapsulated NP cells at pH 7.1 or 6.5, prior to treatment with mechanical compression, does not appear to affect the expression of type VI collagen, α5, β1, α5β1, or fibronectin

Agarose‐encapsulated NP cells stained positive for type VI collagen, α5, β1, α5β1, and fibronectin following 7 days of preculture in standard medium, as described above (Figure 4A). Twenty‐four hours of culture at a pH representative of nondegenerate (pH 7.1) or degenerate (pH 6.5) IVDs, prior to mechanical compression, did not appear to affect the amount of cells expressing type VI collagen, α5, β1, α5β1, or fibronectin (Figure 4B).

## 4 DISCUSSION

Mechanical stimuli are known to be important for IVD cell matrix homeostasis, but the mechanotransduction pathways utilized by disc cells remain poorly understood. Previous mechanical loading studies have predominantly focused on the mechanoresponse of disc cells cultured under standard culture conditions, which fail to mimic the unique and challenging IVD microenvironment. Here, we investigated the influence of acidic pH, one of the most detrimental microenvironmental factors present during IVD degeneration, on the mechanoresponse of human NP cells. We report, for the first time, that the compression response of human NP cells is altered from that of matrix homeostasis at pH representative of nondegenerate tissue (pH 7.1) to matrix catabolism at pH representative of degenerate tissue (pH 6.5). This suggests that acidity‐induced aberrant mechanotransduction is a potential mechanism involved in the progression of IVD degeneration and supports our previous work demonstrating that decreased pH causes a catabolic shift in human NP cell phenotype.^26^

Cells were unresponsive to the compression regime unless cultured for 7 days in standard medium prior to loading. Preculture of encapsulated cells prior to mechanical stimulation has previously been shown to be important in determining whether or not a cell will respond to load.^35^, ^36^, ^37^ Chondrocytes, encapsulated in alginate, showed a greater mechanoresponse (increased matrix proteins and inflammatory cytokines) when precultured for 14 days rather than 24 hours.^35^ Here, we report that there was no change in gene expression in NP cells treated with unconfined compression, unless a 7‐day preculture period was included. This suggests that cells require a preculture period in order to be mechanoresponsive to compressive forces, as shown in chondrocytes previously.^37^ Our IHC analysis revealed that more encapsulated cells expressed type VI collagen, fibronectin, and α5β1 integrins following 7 days of preculture, suggesting that the preculture period promoted the formation of a pericellular matrix. The importance of a pericellular matrix in enabling mechanotransduction has been previously demonstrated.^35^, ^36^, ^37^ Our data support the notion that cells require a pericellular matrix, in combination with matrix‐binding cell surface receptors, in order to sense mechanical stimuli in their surrounding environment. The type VI collagen staining in our study appeared to be on the surface of the NP cells and was not detected more distal from the cells, as might occur with a more mature/established pericellular matrix,^6^ suggesting increased duration of culture may be needed in order to better mimic the in vivo scenario. In addition to the development of a pericellular matrix, encapsulation of two‐dimensional culture‐expanded NP cells in agarose may result in the regaining of NP cell phenotype, which may otherwise be lost during culture.^38^, ^39^ It is difficult to differentiate between three‐dimensional (3D) culture‐induced regain of NP cell phenotype and de novo pericellular matrix production as contributors for the mechanosensitization of encapsulated NP cells. It seems logical that both phenomena may occur in parallel and are intrinsically linked.

The mechanoresponse of NP cells to compression at a nondegenerate pH (pH 7.1) was increased gene expression of all genes assessed. This increase in both anabolic (AGC and TIMP‐1) and catabolic (MMP3 and ADAMTS5) genes is suggestive of a matrix homeostatic response, whereby ECM is remodeled by the cells in response to load. Physiologically relevant hydrostatic compression of NP cells has been shown to lead to matrix remodeling,^14^ suggesting that the mechanical force administered was within the “normal” physiological range. The matrix homeostatic response to mechanical compression, observed at pH 7.1, was altered when cells were compressed at pH 6.5 (representative of degenerate IVDs), with only MMP3 remaining mechanoresponsive. The loss of mechanoregulation of all anabolic, but not all catabolic, genes suggests that NP cells may switch to a more catabolic phenotype when loaded under acidic conditions, similar to those found during IVD degeneration. Such a mechanoresponse could lead to the progression of IVD degeneration in response to physiological compression. However, the number of genes assessed here is limited, and as such, a broader analysis of the NP transcriptome/proteome could help to understand the pH‐dependent mechanoresponse of NP cells in more detail.

To help elucidate the mechanotransduction pathways operating in compressed NP cells, we assessed the involvement of RGD‐integrins in the NP cell mechanoresponse. We found that the matrix homeostatic mechanoresponse (demonstrated by increased AGC and MMP3 gene expression) in NP cells compressed at pH 7.1 was dependent on RGD‐recognizing integrins for changes in AGC expression only. The involvement of RGD‐integrins in the mechanotransduction pathway of both NP^40^ and AF^19^ cells has been previously reported in response to both compression and cyclic tensile strain, respectively. Le Maitre et al reported an RGD‐integrin dependent decrease in AGC gene expression in NP cells derived from nondegenerate tissue in response to high magnitude mechanical compression.^40^ The tethering of RGD peptides to poly(ethyleneglycol) (PEG) hydrogels was shown to enhance the mechanically induced increase in the matrix expression of encapsulated chondrocytes, suggesting chondrocytes also sense compressive strain through RGD‐recognizing integrins.^41^ While we do not know how the fibronectin produced by the encapsulated NP cells in our system interacts with the agarose hydrogel, there does appear to be mechanotransduction occurring, demonstrated by the compression‐induced changes in gene expression. However, tethering of the fibronectin to the agarose hydrogel may lead to an even greater mechanoresponse.

Our data suggest that different mechanotransduction pathways regulate specific genes. We also report that α5β1 integrins were expressed in cells following the 7 days preculture, which further supports a role for integrins as mechanotransducers in human NP cells.

Interestingly, we found that RGD‐recognizing integrins were not involved in the MMP3 mechanoresponse of NP cells at pH 7.1 or 6.5. This finding suggests that an alternative mechanotransduction pathway may be in operation for MMP3 mechanoregulation, which is not affected by acidic pH. Such findings imply that the RGD‐integrin mechanotransduction pathway may be pH‐sensitive, with genes regulated through this pathway (eg, AGC) becoming mechanoinsensitive under acidic pH conditions representative of degenerate IVDs. In fact, differential integrin involvement has been reported previously, with NP cells from nondegenerate, but not degenerate, discs utilizing RGD‐integrins in mechanoregulation of genes.^19^, ^40^ The acidic pH treatment of nondegenerate NP cells reported in this study may have altered the cellular phenotype toward that of a degenerate disc, resulting in the loss of RGD‐integrin involvement following mechanical load.

In order to address whether culture in acidic pH had affected the expression of RGD‐integrins, or their ligands, we assessed the expression of α5β1 integrins; their individual subunits; and their predominant ligand, fibronectin. We found no difference in the expression of these integrins or their ligand at the different pH tested (pH 7.1 and 6.5), suggesting that the loss of expression of AGC at pH 6.5 was not due to reduced α5β1 or fibronectin expression. However, whether or not α5β1 integrin binding to fibronectin at pH 6.5 is altered remains unknown. Fibronectin conformation is pH‐sensitive, with acidic pH shown to cause extension of the fibronectin polymer.^42^, ^43^ Such alterations to the ligands of integrins could prevent the correct binding between the cell and the matrix, resulting in aberrant mechanotransduction. Acidic pH has been suggested to affect the activation state of integrins, with a study finding that avb3 integrin becomes activated at the cell surface under acidic conditions.^44^ Such acidity‐driven activation of integrins could also result in altered mechanosensitivity, leading to altered signaling and loss of integrin‐mediated mechanoregulation of genes.

Although this is the first study to report the combined effects of altered pH and mechanical load on human disc cells, the combined effect of microenvironmental factors associated with IVD degeneration, including pH and mechanical load, have been considered for bovine disc cells. Neidlinger‐Wilke et al demonstrated a pH‐dependent loading response to hydrostatic pressure, with degenerate disc‐associated pH (pH 6.5) and nondegenerate disc‐associated pH (pH 7.2) resulting in a mechanically induced decrease and increase in AGC gene expression, respectively.^27^ In our system, using human NP cells, we report the opposite effect, with acidic pH preventing the compression‐induced increase in AGC gene expression observed at pH 7.1. Potential reasons for the differential response in pH‐ and load‐dependent AGC regulation between studies include differences in the type of loading administered to the cells (unconfined compression in this study vs high‐magnitude hydrostatic pressure in the study by Neidlinger‐Wilke et al), as well as differences in the 3D culture system (agarose in this study vs alginate gel in the study by Neidlinger‐Wilke et al).^27^ Whether this apparent difference in AGC response to pH and load is due to differences between species (human in this study vs bovine in the study by Neidlinger‐Wilk et al) remains a possibility.^27^ Further studies utilizing both human and other species of animal are required in order to gain a better understanding of the combined effect of stress conditions on disc cells.

Using in vitro culture systems to study cell mechanotransduction enables the complexities of the in vivo situation to be somewhat resolved. However, although the advantages of these systems for the study of complex biological phenomena are well accepted, it is worth noting that such a reductionist approach has its limitations. The 3D encapsulation of cells using agarose cannot fully replicate the microenvironment provided by native tissue, with the subsequent loading regime also having its limitation (relatively short duration [1 hour] and the absence of hydrostatic pressure changes). Allowing cells a preculture period may begin to address some of the differences between the simplicity of the hydrogel and the complexity of native tissue, but interpretation of results using these systems should be undertaken with appreciation of such limitations.

To our knowledge, this is the first study to assess the combined effect of mechanical compression and acidic pH on human NP cells. Our findings indicate the importance of considering more than one environmental factor when investigating how disc cells will respond to external stimuli, with the consideration of multiple environmental parameters (low glucose, low oxygen, high osmolarity) required in future to broaden our understanding of the complete system. Our findings suggest that physiologically relevant mechanical stimuli may become detrimental to disc tissue integrity when the pH is reduced. Understanding how cells perceive and respond to signals of both physical and chemical origin will better help our understanding of mechanobiology in human health and disease. These findings will also help increase our knowledge of the pathogenesis of human IVD degeneration and may have major implications for tissue engineering/cell‐based therapeutic strategies for the treatment of disc degeneration and back pain.
